# From convergence insufficiency to functional reorganization: A longitudinal randomized controlled trial of treatment‐induced connectivity plasticity

**DOI:** 10.1111/cns.70007

**Published:** 2024-08-26

**Authors:** Farzin Hajebrahimi, Ayushi Sangoi, Mitchell Scheiman, Elio Santos, Suril Gohel, Tara L. Alvarez

**Affiliations:** ^1^ Department of Biomedical Engineering New Jersey Institute of Technology Newark New Jersey USA; ^2^ Pennsylvania College of Optometry Salus University Philadelphia Pennsylvania USA; ^3^ Department of Health Informatics Rutgers University School of Health Professions Newark New Jersey USA

**Keywords:** binocular vision, convergence insufficiency, fMRI, resting‐state functional connectivity

## Abstract

**Introduction:**

Convergence Insufficiency (CI) is the most prevalent oculomotor dysfunction of binocular vision that negatively impacts quality of life when performing visual near tasks. Decreased resting‐state functional connectivity (RSFC) is reported in the CI participants compared to binocularly normal control participants. Studies report that therapeutic interventions such as office‐based vergence and accommodative therapy (OBVAT) can improve CI participants' clinical signs, visual symptoms, and task‐related functional activity. However, longitudinal studies investigating the RSFC changes after such treatments in participants with CI have not been conducted. This study aimed to investigate the neural basis of OBVAT using RSFC in CI participants compared to the placebo treatment to understand how OBVAT improves visual function and symptoms.

**Methods:**

A total of 51 CI participants between 18 and 35 years of age were included in the study and randomly allocated to receive either 12 one‐hour sessions of OBVAT or placebo treatment for 6 to 8 weeks (1 to 2 sessions per week). Resting‐state functional magnetic resonance imaging and clinical assessments were evaluated at baseline and outcome for each treatment group. Region of interest (ROI) analysis was conducted in nine ROIs of the oculomotor vergence network, including the following: cerebellar vermis (CV), frontal eye fields (FEF), supplementary eye fields (SEF), parietal eye fields (PEF), and primary visual cortices (V1). Paired t‐tests assessed RSFC changes in each group. A linear regression analysis was conducted for significant ROI pairs in the group‐level analysis for correlations with clinical measures.

**Results:**

Paired t‐test results showed increased RSFC in 10 ROI pairs after the OBVAT but not placebo treatment (*p* < 0.05, false discovery rate corrected). These ROI pairs included the following: Left (L)‐SEF–Right (R)‐V1, L‐SEF–CV, R‐SEF–R‐PEF, R‐SEF–L‐V1, R‐SEF–R‐V1, R‐SEF–CV, R‐PEF–CV, L‐V1–CV, R‐V1–CV, and L‐V1–R‐V1. Significant correlations were observed between the RSFC strength of the R‐SEF–R‐PEF ROI pair and the following clinical visual function parameters: positive fusional vergence and near point of convergence (*p* < 0.05).

**Conclusion:**

OBVAT, but not placebo treatment, increased the RSFC in the ROIs of the oculomotor vergence network, which was correlated with the improvements in the clinical measures of the CI participants.

## Graphical abstract

Office‐based Vergence and Accommodative Therapy, but not placebo therapy, improves the resting‐state functional connectivity in the regions of the oculomotor vergence network which is correlated with the improvements in the clinical measures using in the assessment and diagnosis of CI.

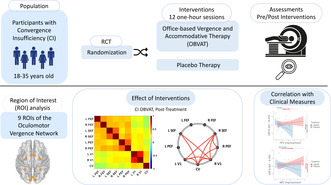

## 1 INTRODUCTION

Convergence Insufficiency (CI) is the most prevalent oculomotor dysfunction of binocular vision in adolescents and adults.^1^, ^2^ The primary clinical signs of CI include a receded near point of convergence, reduced positive fusional vergence at near distance (40 cm), and more exodeviation at near distances compared to far (6 m). CI participants often experience blurred and / or double vision, visual fatigue, headaches, and difficulty concentrating when performing near‐visual tasks such as reading or using digital devices.^3^, ^4^ These symptoms worsen during activities that require prolonged near‐focused tasks, such as reading or using digital devices, which can negatively affect the quality of life for individuals engaged in extended periods of close visual work.^5^, ^6^

Several studies have reported similar effectiveness of therapeutic interventions, including office‐based vergence and accommodative therapy (OBVAT), in treating the visual symptoms and functionality in participants with CI,^7^, ^8^, ^9^, ^10^, ^11^, ^12^, ^13^, ^14^, ^15^, ^16^ as also reported in a Cochrane Library network meta‐analysis.^17^ Recently, the results of a randomized controlled trial (RCT) called the convergence insufficiency neuro‐mechanism in adult population study (CINAPS) showed the effectiveness of OBVAT in a sufficiently powered sample of CI participants and reported that about 75% were classified as either successful or improved after OBVAT.^18^

Previous results of the CINAPS^19^ and previous small‐sampled task‐related functional magnetic resonance imaging (fMRI) studies have shown the possible underlying neural mechanism of CI in mediating vergence eye movements with changes in the blood oxygenation level dependent (BOLD) signal.^20^, ^21^, ^22^, ^23^, ^24^, ^25^ These studies have also shown that functional activation in the specific brain regions is captured after stimulating symmetrical vergence steps compared to sustained midline fixation within an MRI scanner. These prior studies have identified the following brain regions within the oculomotor vergence network as Region of Interest (ROI): frontal eye fields (FEF), supplementary eye field (SEF), parietal eye fields (PEF), cerebellar vermis (CV), and primary visual cortex (V1). These ROIs are shown to be functionally and reliably activated during the vergence responses after a vergence eye movement‐inducing stimulus inside the MRI scanner.^26^ However, previous literature has not addressed how these brain hubs communicate regarding resting‐state functional connectivity (RSFC) for a therapeutic intervention of CI. Importantly, other neural mechanism(s) may be present to explain how the OBVAT remediates CI further. Future RCTs can be designed to investigate how therapeutic interventions can modify the underlying neuro‐mechanism and clinical manifestation of CI, which may lead to improved personalized point‐of‐care interventions. These RCTs can be assessed via evidence‐based brain measurements and enhance treatment effectiveness.

FMRI is a valuable non‐invasive tool for understanding the neuro‐mechanism of therapeutic interventions that could potentially lead to neuroplasticity or modifications in the neural networks. To understand how treatments affect the brain dynamics in CI, some pilot longitudinal studies with small sample sizes have shown the effectiveness of vision rehabilitation using the changes in the task‐modulated activation after the treatment,^27^, ^28^ as well as the task‐based “coactivation” in the vergence system of the brain.^25^ Resting‐state fMRI (rs‐fMRI) measures slow oscillations in the BOLD signal and its advantage lies in the fact that the participants do not engage in any specific task.^29^, ^30^ Hence, rs‐fMRI has the potential for greater consistency within scientific investigations. Rs‐fMRI shows how different brain regions communicate with each other and can identify the neurovascular mechanisms of visual dysfunctions such as strabismus and amblyopia,^31^, ^32^, ^33^, ^34^, ^35^ exotropia^36^ and other brain‐related diseases such as psychiatric disorders, Parkinson's Disease, and multiple sclerosis.^37^, ^38^, ^39^, ^40^, ^41^, ^42^ Of various methods employed in rs‐fMRI, RSFC can evaluate the plasticity after an intervention^43^ and has been frequently used to study the effectiveness of the treatments in different diseases such as amblyopia,^44^ traumatic brain injury,^45^ Parkinson's Disease,^46^ stroke,^47^ and many other neurological diseases and disorders. Due to the ease of acquisition and standardization of rs‐fMRI protocols compared to task‐related stimulus‐induced protocols, it is promising that rs‐fMRI can be translated to clinical settings in the future.^48^ In this regard, rs‐fMRI and RSFC can be an outcome measurement in a longitudinal RCT to remediate disease or dysfunction. RSFC can potentially serve as a non‐invasive biomarker of diseases and dysfunctions.

Prior research from the CINAPS showed a decreased RSFC in the CI participants compared to binocularly normal control participants. The decreased RSFC in CI participants was observed in the visual, frontoparietal, and default mode networks and was clinically correlated with CI diagnostic signs. Therefore, the results suggest a significant interrelation between altered RSFC and clinical measurements in CI.^49^ To date, prior studies have not yet longitudinally investigated the impact of OBVAT on CI using RSFC. As a part of the CINAPS, this RCT aimed to compare the effect of OBVAT on RSFC in CI participants to an age‐matched group of CI participants undergoing placebo treatment. The present study will test the hypothesis that RSFC improvements will be correlated to the improvement in clinical signs and symptoms observed post‐OBVAT compared to baseline measurements.

## 2 METHODS

### 2.1 Study design and participants

This study was a double‐masked, placebo‐controlled, randomized interventional clinical trial, and was part of the CINAPS. The protocol of the CINAPS followed the Declaration of Helsinki, reviewed and approved by the Institutional Review Board (IRB) of the New Jersey Institute of Technology and Rutgers University, and registered on ClinicalTrials.gov (Identifier: NCT03593031). Enrollment began in August 2015 and ended on April 2018.

**FIGURE 1:**
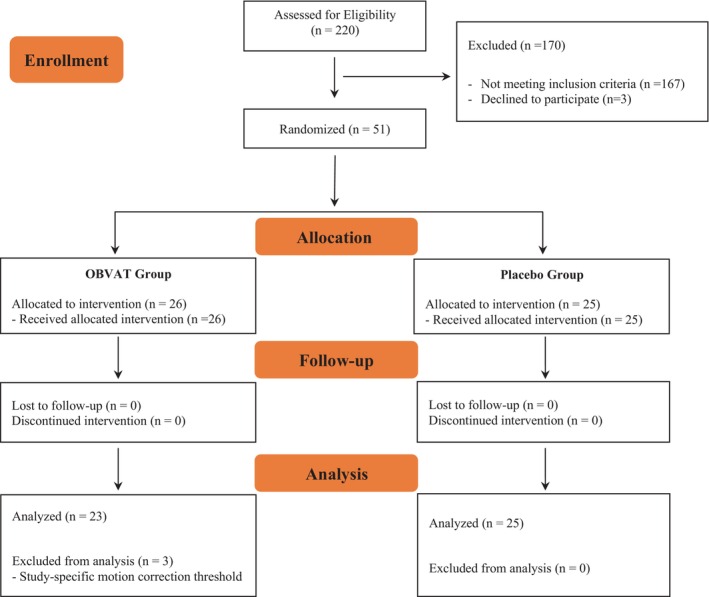
The CONSORT flowchart of the study. OBVAT, office‐based vergence and accommodative therapy.

A written informed consent was obtained from all participants. The details related to the participant selection, study design, and methodology of the CINAPS are explained extensively in a previous paper.^50^ Briefly, 51 participants between 18 and 35 years who had been diagnosed with symptomatic CI were included in the study. Participants must have a visual acuity of 20/25 or better with proper refractive correction if needed, normal local stereopsis of 70 seconds of arc, and global stereopsis of 500 seconds of arc or better to be included in the study. Participants with a history of head injury and concussion or any neurological or retinal disease were excluded from the study. Following inclusion in the study, participants were randomly allocated to receive either OBVAT or office‐based placebo therapy. All participants underwent clinical assessments and rs‐fMRI at baseline and outcome assessments. Treatments in each group consisted of 12 office‐based treatment sessions, twice a week, for 1‐hour sessions over a 6‐to‐8‐week duration. Additionally, all participants were instructed to perform home reinforcement for three sessions per week. Each home session lasted for about 10 minutes. The CONSORT flowchart of the study is shown in Figure 1.

### 2.2 Randomization and masking

Randomization was applied using a random number generator function from a MATLAB program. Patients were randomly allocated to two treatment arms. Investigators performed an outcome examination, and participants were masked and unaware of their group allocation. All participants were aware that they would be randomly allocated to either of the groups and did not learn their group allocation until the end of the study. The CI participants allocated to receive placebo treatment had the option to undergo OBVAT at the end of the study. This procedure was explained in the consent forms.

### 2.3 Interventions

The complete details of the treatment protocols used are explained in previous publications.^18^, ^50^ Patients in both groups received 12 one‐hour office‐based therapy sessions delivered by a study‐certified therapist over a period of 6 to 8 weeks (1 to 2 sessions per week). Patients were instructed to perform 10 minutes of computer‐based home exercises 3 days per week on days not participating in the office‐based therapy. OBVAT consisted of procedures to improve positive and negative fusional vergence, near point of convergence, and accommodative function. OBVAT procedures progressed in difficulty over the 12 sessions. Placebo treatment simulated OBVAT without stimulating vergence or substantial accommodative changes. Details of these therapies can be found in a previous publication.^50^

### 2.4 Clinical assessment for diagnosis of CI

All participants were examined by a study‐certified optometrist (co‐author MS). The clinical assessments for CI diagnosis included positive fusional vergence (PFV) at near (40 cm), near point of convergence (NPC), the difference between the horizontal phoria at near (40 cm), and far (6 m, Diff‐Phoria) and the Convergence Insufficiency Symptom Survey (CISS).^2^, ^4^ Near point of convergence is measured from the bridge of the nose and is the distance along midline when a participant reports seeing double vision of a high acuity target. Positive fusional vergence is measured with a prism bar that has a resolution of 2 prism diopters (∆) from 2 to 20∆ and a resolution of 5∆ from 20 to 45∆. Positive fusional version is the participant's perception of blur through a prism when viewing a high acuity target 40 cm from midline. If blur is not perceived, then positive fusional vergence is measured as the prism when the participant reports double vision. Detailed procedures of the clinical optometric assessments are described in the CINAPS design paper^50^ and the convergence insufficiency treatment trial.^51^, ^52^ Screened participants were diagnosed as having symptomatic CI using the following criteria: (1) reduced PFV at near defined as either failing Sheard's criterion or having a measurement of less than 15 prism diopters (∆) base‐out at near (40 cm); (2) receded NPC of ≥6 cm; (3) exophoria at near at least 4∆ greater than at far (6 m); and (4) a symptom score of ≥21 on the CISS for young adults.

### 2.5 Data acquisition and preprocessing

#### 2.5.1 Data acquisition

Data acquisition was performed at baseline and outcome for both intervention groups (OBVAT and placebo treatment). A 3T Siemens TRIO (Siemens Medical Solutions, USA) with a 12‐channel head coil was used. The experimental protocol, nature of the scanning, and safety precautions were explained to the participants prior to each scan. Each participant was instructed to lie still, and the researchers emphasized not moving their head during the scanning, staying awake, and looking at a small square on the MR screen. All participants were asked to remove metal or other ferrous materials before entering the scanning room. Spongy pads were placed around the head inside the head coil to minimize the head motion.

#### 2.5.2 Scanning parameters

A magnetization‐prepared rapid acquisition gradient‐echo (MPRAGE) sequence was used to acquire a high‐resolution anatomical image. Patients were asked to close their eyes during the anatomical scanning. The parameters of the MPRAGE sequence were the following: time of repetition (TR) = 1900 ms, time of echo (TE) = 2.52 ms, field of view (FOV) = 256 mm, flip angle = 9°, total number of acquired slices = 176, and voxel resolution = 1.0 × 1.0 × 1.0 mm. A single echo planar imaging (EPI) sequence was used to obtain functional volumes with the following parameters: TR = 2000 ms, TE = 13 ms, FOV = 192 mm, flip angle = 90°, number of volumes = 150, total number of acquired axial slices = 53, and voxel resolution = 3.0 × 3.0 × 3.0 mm.

#### 2.5.3 Data preprocessing

The data preprocessing pipeline was similar to the procedure in the previous research.^49^ It utilized the following toolboxes: Statistical Parametric Mapping (SPM) version 12 (https://www.fil.ion.ucl.ac.uk/spm/software/) in MATLAB version 2023a, Analysis of Functional Neuroimages toolbox (AFNI)^53^ (http://afni.nimh.nih.gov/afni/), and FMRIB's Software Library (FSL) version 6.0.6.2^54^ (https://fsl.fmrib.ox.ac.uk/fsl/fslwiki/). The first five time points of each rs‐fMRI dataset were removed to eliminate the effects of T1‐relaxation. The functional data were aligned to the MPRAGE data and centered on the Anterior Commissure (AC) as a reference. Motion correction for the functional data was performed using SPM's realign function. Each volume of the functional data was aligned to the mean of the volumes. Next, functional data were co‐registered with the anatomical data. Probability maps were used to segment the anatomical data into gray matter (GM), white matter (WM), and cerebrospinal fluid (CSF) images. A deformation field was derived during the segmentation step to transform every functional data into the Montreal Neurological Institute (MNI) standard space. WM and CSF masks were created using the probability maps of WM and CSF thresholded at *p* > 0.95 and further used to extract the CSF and WM time series from the functional data in the MNI space. The first five principal components from the CSF and WM time series were extracted using a principal component analysis. A General Linear Model (GLM) with 34 regressors (six motion parameters calculated in the realignment steps, six autoregressive versions of the motion parameters and 12 quadratics of these motion parameters,^55^ five WM, and five CSF components^56^) was used to regress out the physiological and motion‐based artifacts, and the CSF and WM covariances from the functional data was used to regress out the physiological and motion‐based artifacts, and the CSF and WM covariances from the functional data. Temporal filtering was applied within the 0.01 and 0.1 Hz frequency bands and spatial smoothing was applied with a Gaussian kernel of 6 mm full width at half maximum (FWHM).

### 2.6 Data analysis

#### 2.6.1 Head motion analysis

Motion parameters were recorded in six directions, and frame‐wise displacement was calculated for each functional dataset. Data with an frame‐wise displacement greater than half of each functional voxel dimension (1.5 mm), were excluded from the analysis.^57^, ^58^ To identify whether there were any systematic differences in head motion between the two groups, frame‐wise displacement parameters were compared at baseline and after the therapeutic interventions. The Shapiro–Wilk test assessed the normality of the frame‐wise displacement parameters, revealing a non‐normal distribution. Therefore, non‐parametric tests were applied to compare the frame‐wise displacement parameters.

### 2.7 Resting‐state functional connectivity analysis

#### 2.7.1 ROI analysis

Region of Interest (ROI) analysis was employed to discover the effectiveness of each treatment. This investigation builds upon prior research identifying the ROIs responsible for the oculomotor vergence network. Previous studies have shown that stimulating symmetrical vergence steps, as opposed to sustained midline fixation within an MRI scanner, results in functional activation in nine specific ROIs associated with the oculomotor vergence network.^19^, ^20^, ^21^, ^22^, ^23^, ^24^, ^25^ These regions have been reliably activated during vergence responses to a vergence eye movement‐inducing stimulus inside the MRI scanner with a high intraclass correlation coefficient.^26^ The nine ROIs included in the ROI analysis were the followings, each specified with the respective center MNI coordinates: left FEF (MNI: −28 −6 48), right FEF (MNI: 28 −6 52), left SEF (MNI: −6 8 50), right SEF (MNI: 6 8 50), left PEF (MNI: −30 −48 50), right PEF (MNI: 30 −48 50), left V1 (MNI: −10 −88 6), right V1 (MNI: 10–88 6), and CV (MNI: −2 −78 −22).

For each ROI, center MNI and voxel coordinates were used to create a sphere mask with a radius of 6 mm within the MNI template. RSFC time series were extracted in each ROI mask from the preprocessed fMRI data for each dataset. A pair‐wise correlation was calculated for every pair of ROIs to calculate the RSFC between each ROI, and a 9 × 9 correlation coefficient matrix was created for each dataset. The distribution of RSFC measures across participants must be approximately normally distributed to utilize standard parametric tests to assess hypotheses at the group‐level. Due to the restricted range of r values in the correlation matrices (−1 to 1), possible bias can occur; hence, Fisher's r‐to‐z transformation was applied to the correlation matrices to calculate Z values for each ROI pair, thereby improving data normality. This step is common and crucial in studying the changes in functional connectivity research.^59^ Furthermore, the Shapiro–Wilk test was used to verify the normality empirically, and none of the 36 ROI pairs showed deviation from a normal distribution (*p* < 0.05, FDR corrected for multiple comparisons).

#### 2.7.2 Group‐level analysis

Several statistical measures were performed to investigate the potential impact of the neural substrates from OBVAT compared to placebo treatment to assess treatment and group differences. First, paired t‐tests were used to compare the RSFC strength before and after OBVAT and placebo treatment in MATLAB. Next, a mixed model analysis was employed to discover the time × group interaction in R using the Jamovi Software.^60^ RSFC values of the OBVAT and placebo treatment at baseline and outcome were the inputs for this model. To account for the multiple comparisons, all *p* values were adjusted using the False Discovery Rate (FDR) method.^61^ Only datasets with both the time‐points data (pre‐ and post‐treatment data sets) were included in the paired t‐tests and mixed model analysis.

#### 2.7.3 Correlation analysis between RSFC and clinical measures

A linear regression model was implemented in MATLAB to analyze the correlation between the RSFC strength and clinical measures. This model was applied to each of the ROI pairs that had significant results in the group‐level analysis. For this reason, the RSFC strength of the significant ROI pairs and clinical measures were first obtained. Subsequently, the differences between the baseline and outcome values for the RSFC strength and clinical measures (i.e., improvements in the clinical measures) were calculated. These differential values for the clinical measures and the group variables were then utilized as input for the regression model. The output of the model included the RSFC variables. The regression analysis aimed to identify whether the correlations between the RSFC and the clinical measures significantly differ between the OBVAT and placebo treatment groups. Therefore, compared to the correlation analyses in each group, which would show the possible positive or negative non‐zero correlations between the RSFC and clinical measures, the current analysis also included the interaction term between the group and the clinical measures. These interaction terms account for the comparisons between the slopes in each group for every correlation between the RSFC and clinical measures. Therefore, the interaction term would depict whether the correlation between RSFC and clinical measures significantly differs between groups. Consequently, the interaction term between the group and the clinical measures and the significance value for the differences between the slopes were extracted from the model. Additionally, correlation coefficient values for each group were extracted separately.

## 3 RESULTS

### 3.1 Demographics and clinical characteristics

Fifty‐one participants with the diagnosis of symptomatic CI were included in this study. After the initial screening, participants were randomly allocated to OBVAT (*n* = 26) and placebo treatment (*n* = 25) groups. The datasets for three participants in the OBVAT group were removed from the further RSFC analysis due to study‐specific motion criteria. The final dataset analyzed in each group were 23 participants in the OBVAT group (10 female) and 25 participants in the placebo treatment group (13 female). Table 1 shows the demographic information of each group at baseline before inclusion in the interventions, as well as the clinical characteristics and head motions. Normality testing using the Shapiro–Wilk test on clinical parameters revealed non‐normality for age, PFV, NPC, and Diff‐Phoria. The Mann–Whitney U test was used to compare groups at baseline for these non‐normally distributed parameters. An independent Samples t‐test was used for the CISS values at baseline. Both groups were similar in demographics and clinical measures at baseline (*p* > 0.05).

**TABLE 1:** Demographics and group differences at baseline.

	OBVAT *n* = 23 M ± SD	Placebo *n* = 25 M ± SD	Between group difference
Demographics			
Age (years)	21.22 ± 3.73	20.6 ± 3.63	*U* = 261, *p* = 0.575^a^
Sex	10 ♀ 13 ♂	13 ♀ 12 ♂	*χ* ^2^(1) = 0.349, *p* = 0.555^b^
Clinical			
PFV (∆)	10.43 ± 3.01	10.16 ± 3.36	*U* = 280, *p* = 0.873^a^
NPC (cm)	10.13 ± 3.51	10.42 ± 3.28	*U* = 265, *p* = 0.648^a^
Diff‐Phoria (∆)	−6.57 ± 2.61	−5.6 ± 2.71	*U* = 252, *p* = 0.463^a^
CISS (points)	32.83 ± 7.87	35.44 ± 6.43	*t*(46) = −1.26, *p* = 0.212^c^
Head motion correction			
Frame‐wise displacement (mm)	0.056 ± 0.024	0.066 ± 0.04	*U* = 251, *p* = 0.461^a^

*Note*: ♀: Female; ♂: Male.

Abbreviations: CISS, convergence insufficiency symptom survey; Diff‐Phoria, differences of the horizontal phoria plano in near and far; M, mean; NPC, near point of convergence; OBVAT, office‐based vergence and accommodative therapy; PFV, positive fusional vergence; SD, standard deviation.

^a^ Mann–Whitney *U* test.

^b^ Chi‐square test.

^c^ Independent samples *t*‐test.

### 3.2 Head motion

Normality testing using the Shapiro–Wilk test on the frame‐wise displacement parameters revealed non‐normality. Hence, non‐parametric tests were used to assess differences in frame‐wise displacement parameters. A Mann–Whitney U test showed no significant difference between the two groups at baseline (*p* > 0.05, Table 1). Additionally, the Wilcoxon test showed no significant difference in frame‐wise displacement before and after treatment within the OBVAT group (*p* > 0.1) and the placebo treatment group (*p* > 0.9).

### 3.3 Effect of interventions

**FIGURE 2:**
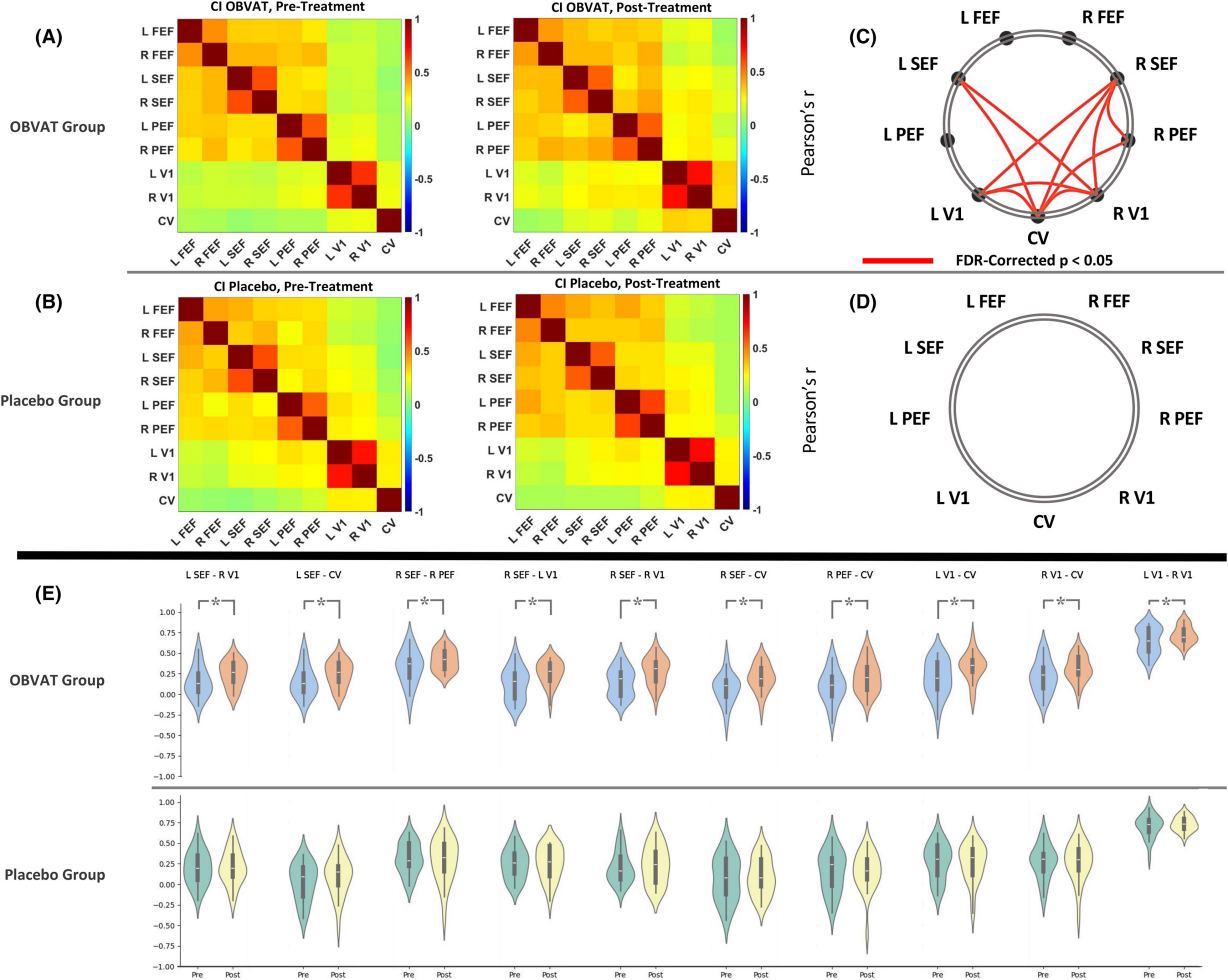
RSFC changes after OBVAT versus placebo treatment. (A) Correlation coefficient heatmap matrices for OBVAT group before and after the treatment. Each symmetric heatmap matrix shows 36 possible correlations below or above the diagonal; (B) Correlation coefficient heatmap matrices for placebo group before and after the treatment; (C) ROI pairs with a significant change in the RSFC after the OBVAT. Red line indicates significant RSFC between the ROIs in the OBVAT group's correlation matrix after the treatment compared to the correlation matrix before the treatment; (D) No significant change in the RSFC of the ROIs was found in the placebo group after the treatment; (E) The strength of the group‐level average RSFC in the ten significant ROI pairs in the OBVAT group and in the placebo group (for comparison purposes). CV, cerebellar vermis, FEF, frontal eye field; FDR, false discovery rate; L, left; OBVAT, office‐based vergence and accommodative therapy; PEF, parietal eye field; R, right; SEF, supplementary eye field; V1, primary visual cortex.

The paired t‐test results showed an increase in RSFC in 10 ROI pairs after the treatment in the OBVAT group (*p* < 0.05, FDR corrected). These ROI pairs included L‐SEF–R‐V1, L‐SEF–CV, R‐SEF–R‐PEF, R‐SEF–L‐V1, R‐SEF–R‐V1, R‐SEF–CV, R‐PEF–CV, L‐V1–CV, R‐V1–CV, and L‐V1–R‐V1. No significant results were found in the paired t‐tests in the placebo treatment group (*p* > 0.05, FDR corrected). Figure 2A shows the heatmaps for the mean correlation coefficient matrices for OBVAT and placebo treatment groups before and after the treatment. Additionally, ROI pairs with a significant change in the RSFC after the OBVAT are shown in Figure 2A. The strength of the group‐level average RSFC in the 10 significant ROI pairs in the OBVAT group is shown in Figure 2B. For comparison purposes, the group‐level average RSFC strength for the same ROI pairs is given in Figure 2B.

### 3.4 Comparison between interventions

The time × group interaction was utilized to assess the impact of vision therapy on both groups over time and to determine whether a differential effect existed between them. This analysis was conducted across all ROI pairs, with subsequent correction for multiple comparisons using FDR at *p* < 0.05. After correction for multiple comparisons, the time × group interaction test revealed no significant differences between the OBVAT and Placebo groups (*p* < 0.05 FDR corrected). However, uncorrected results showed a significant time × group interaction in favor of the OBVAT group in the RSFC of the L‐V1–CV ROIs (*p* = 0.032). Post hoc analysis showed no significant difference between groups before treatment (*p* > 0.05), with the OBVAT group showing a significant increase in RSFC of these ROIs post‐treatment (F(46) = 7.105, *p* = 0.0105).

### 3.5 Correlation with clinical measures

**FIGURE 3:**
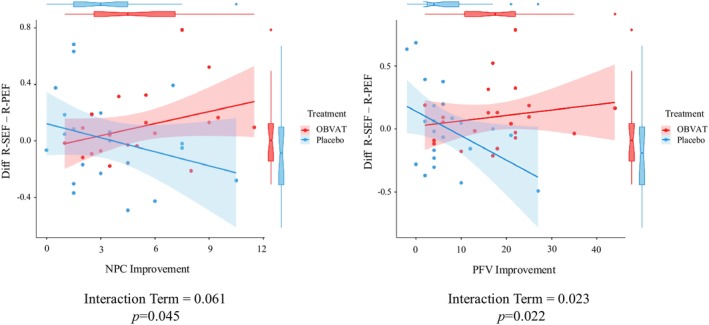
Correlation of the RSFC strength before and after treatment with clinical measures in OBVAT versus placebo treatment groups. Diff, difference value; NPC, near point of convergence; OBVAT, office‐based vergence and accommodative therapy; R‐PEF, right parietal eye field; PFV, positive fusional vergence; R‐SEF, right supplementary eye field.

A regression model compared the correlation of the RSFC strength for baseline and outcome measurements with the clinical visual measures for the OBVAT and placebo treatment groups. Three participants in the placebo treatment group and one in the OBVAT group had missing clinical measurements and were removed from the correlation analysis. The final correlation analysis was conducted with 22 participants in each group. A significant interaction was observed between the RSFC strength of the R‐SEF–R‐PEF ROI pair and the clinical measures of PFV (*Interaction Term* = 0.023; *p* = 0.022) and NPC (*Interaction Term* = 0.061; *p* = 0.045). Increased RSFC of the R‐SEF–R‐PEF was significantly correlated with improved clinical measures in both PFV and NPC in the OBVAT group. The placebo treatment group showed the opposite pattern (Figure 3). The group‐level correlation coefficients in the OBVAT and placebo treatment groups were 0.185 and − 0.433 for PFV and 0.346 and − 0.287 for NPC, respectively. No other significant correlation was found between the RSFC strength of the rest of ROIs with clinical measures (*p* > 0.5).

## 4 DISCUSSION

This study was designed to assess how OBVAT may affect the neuronal system of CI participants and whether this effect is different compared to the placebo treatment. A second aim was to determine whether the difference between the intervention arms is correlated with the clinical measures commonly used to diagnose participants with CI. While clinical studies have shown the effectiveness of the OBVAT in remediating CI, understanding its effect on the underlying resting state of the brain can further elucidate how this treatment facilitates neuroplasticity. The findings can highlight the potential applications of OBVAT to improve the visual function of other neurological dysfunctions such as traumatic brain injury.^62^, ^63^ The results of the current study support that OBVAT, but not placebo treatment, improves the RSFC in the ROIs of the oculomotor vergence network, which is correlated with the improvements in the two clinical measures used in the assessment and diagnosis of CI. The correlation analysis with clinical measures performed in the current study reveals that the relationship between RSFC and clinical measures is significantly different between the two treatment groups regarding improvements in PFV and NPC. Specifically, the OBVAT group positively correlated with clinical measures, indicating that increased RSFC strength was associated with greater clinical improvements. Conversely, the placebo group showed a negative correlation with clinical measures, suggesting an opposite pattern where changes in RSFC were not associated with clinical improvements. These results highlight the differences between the OBVAT and placebo treatments, underlining the neuroplasticity of the therapeutic intervention in brain‐behavior relationships.

A previous study emphasized that CV, V1, PEF, SEF, and FEF are functionally active during a vergence task in humans, suggesting their potential use in fMRI studies related to vergence oculomotor responses.^26^ The current study showed an increase in RSFC of the primary brain hubs responsible for executing vergence movements following OBVAT. Among the 10 pairs of ROIs showing significantly increased RSFC post‐OBVAT, CV was involved in five of these pairs. R‐SEF and R‐V1 were associated with four pairs, while L‐V1 was linked to three pairs. L‐SEF and R‐PEF were involved in two pairs each. Interestingly, L/R‐FEF and L‐PEF did not exhibit significant changes in RSFC with the other six ROIs of the oculomotor vergence network following OBVAT. It can be inferred from the results that OBVAT is mostly an oculomotor rehabilitation that improves how the motor hubs of the oculomotor vergence network mediate vergence eye movements. These results can be linked with the corresponding improvements in performing visual functions measured clinically in CI participants after OBVAT. However, how OBVAT may affect cognitive functions in participants with CI and the relationship with specific brain regions responsible for different cognitive functions is beyond the scope of this study. Further investigation to understand the cognitive burden of CI and the effectiveness of OBVAT on cognitive functions in CI participants is warranted.

The significant correlation of the increased RSFC of the R‐SEF–R‐PEF with two primary diagnostic criteria for CI (NPC and PFV) is clinically impactful for visual scientists, specifically and more broadly for neuroscientists. SEF and PEF are both regions critical for visual attention and are active in visual attention tasks.^64^, ^65^ Clinical improvements after OBVAT are correlated with increased RSFC of the R‐SEF and R‐PEF, supporting the effects of OBVAT in strengthening the links with neural substrates of attention in the brain. Here, OBVAT strengthens traces of the cognitive functions related to visual attention activities. This trend is not present in placebo treatment, showing the impact of OBVAT in mediating cognitive brain functions besides its main role in rehabilitating oculomotor functions. Tests of cognitive functions for the CI participants within this study were not part of the study design. Hence, this observation necessitates further investigation to test CI participants' oculomotor and cognitive functions.

Significant results in the correlation with clinical measures were observed in the right hemisphere (R‐PEF–R‐SEF). Studies have highlighted that attention regions in the right hemisphere exhibit tonic activity during rest periods and without visual stimuli.^64^ Similar studies have also emphasized the dominant role of the right parietal lobe in visual attention compared to the left hemisphere.^66^ Although the current study cannot provide information regarding the endogenous or exogenous type of attention that might be strengthened after OBVAT, the results support that the increased RSFC of regions on the right hemisphere may explain this tonic activity. In other words, OBVAT has modulated the right hemisphere to anticipate forthcoming visual attention tasks. The anticipatory behavior of the right brain regions may be linked to their increased functional activity after performing visual attention and convergence tasks. However, further studies are needed to prove this hypothesis by correlating the task activations with RSFC of the same regions. Of note, this study's results do not provide information about the directionality of the increased RSFC between R‐SEF and R‐PEF. Such future analysis can provide information on the type of attention that OBVAT may improve in participants with CI. Collectively, improvements in PFV and NPC measured clinically, which show the improved oculomotor function of the CI participants, may be associated with improved attention based on the improvements in brain hubs responsible for endogenous/exogenous attention. This can overall be linked to activities of daily living that need combined attention and convergence, such as reading and using digital devices within near range (less than 40 cm).

Studies have shown that the cerebellum, specifically the CV, partly mediates vergence eye movements. Lesions in the CV can lead to dysfunctional vergence responses in humans.^20^, ^67^ Additionally, research has highlighted the cerebellum's role in adapting the vergence system for improved binocular vision.^68^ In our study, among the nine ROIs examined, within‐group comparisons in the OBVAT group revealed that the CV exhibited the highest number of significant changes in RSFC with other ROIs. The increased RSFC of the CV suggests that OBVAT positively affects the RSFC of the CV with other hubs of the oculomotor vergence network in CI participants. Given the CV's role in mediating vergence, the increased RSFC of the CV may be associated with improved clinical vergence performance in participants with CI after OBVAT.

Primary visual cortices (V1) play a crucial role in binocular vision in humans.^69^, ^70^ Electroencephalography studies have shown brain activity signals in cortical regions, including the frontal and cerebellar regions, that precede human convergence movements.^71^ Thus, V1 activity can precede the execution of vergence movements, with subcortical regions playing a more dominant role during the actual movements.^71^ The increased RSFC of sensory hubs, including R/L‐V1, with the rest of the oculomotor vergence network observed in this study, suggests strengthened RSFC of the sensory regions with each other, with the cerebellum, and with motor hubs of the oculomotor vergence network post‐OBVAT. Right and left V1 showed significantly increased RSFC when interacting with each other and other areas of the oculomotor vergence network, contributing to four and three significant pairs, respectively. The increased RSFC of the V1 regions with the cerebellum and frontoparietal regions may indicate anticipatory behavior of the sensory components of the oculomotor vergence network for upcoming movements requiring convergence. Additionally, increased V1 RSFC can be seen as an improvement in binocular vision following OBVAT. The results of this study also indicated a significant time × group interaction in favor of the OBVAT group in the RSFC of the L‐V1–CV ROIs compared to the placebo group. This finding emphasizes a significant increase in the RSFC of these ROIs in the OBVAT group post‐treatment, whereas the placebo group did not show such an increase. While these results were not significant after correction for multiple comparisons, they are clinically meaningful because they provide an understanding of the neuroplasticity evoked from OBVAT but not placebo treatment for those with CI.

Finally, although this study's results did not show significant differences in the RSFC of the FEF, the increased RSFC in other brain regions, such as SEF and PEF, may potentially indicate their favorable impact on the enhanced functionality of the FEFs during the performance of daily tasks necessitating convergence. The FEF receive significant projections from SEF and PEF that contribute to the precise functioning of the FEF.^72^, ^73^ Since the results showed a dominance of the increased RSFC of the SEF and PEF and this connectivity was modified with clinical measures, it can be concluded that increased RSFC of the SEF and PEF may have contributed to enhanced communication with the FEF after OBVAT, and altogether this leads to clinical improvements in CI participants after OBVAT.

This study is the first to investigate the effectiveness of OBVAT in CI participants using the RSFC. However, there are some limitations. First, this study is ROI‐based, and results are limited to the current ROIs examined. Further studies with whole brain parcellation may further help to understand OBVAT's impact on the entire brain RSFC and network reorganization in CI. However, considering the challenges encountered in every RCT, such a study would need precise power calculation, considering the correction for multiple comparisons for many ROIs. Second, although the inferences used here can imply the mechanism of the effectiveness of OBVAT in modulating different brain regions, they do not provide information on the causality.^74^ Further studies with causality analysis of RSFC in the CI participants after OBVAT are warranted. Third, the study's result supports the static RSFC between the ROIs, if the RSFC does not change over time. Further analysis can be performed to understand the dynamic RSFC between ROIs in a specific time scale. Although it is a challenging and debatable topic to choose the accurate time‐shifting windows and beyond the scope of this study, that kind of analysis would also aid in understanding how the RSFC can change during time in the resting states and after performing a vergence task. Finally, due to the nature and priorities of the CINAPS, the resting‐state sequence was planned after task‐related scans and a structural scan. Patients may have experienced potential fatigue, but this impact is negligible considering the young age of the population in the study.

The results of this study indicate that OBVAT leads to improved functioning of the oculomotor vergence network in performing daily life tasks that need involuntary vergence movements in their nature. Further studies correlating the RSFC in the oculomotor vergence network and the task‐related functional activity in the same regions are necessary to understand the task‐rest interpretability of the oculomotor vergence network and how OBVAT can modulate this. The results of the current study can also be used as a foundation for further studies investigating vergence‐related problems in other clinical populations such as concussion,^75^ traumatic brain injury,^62^, ^63^, ^76^ and Parkinson's Disease.^77^, ^78^, ^79^ A better understanding of the underlying mechanism of how OBVAT changes the brain's neural networks may provide valuable information that eye care professionals can use to improve the management of oculomotor problems in different clinical populations based on their specific pathophysiology.

## FUNDING INFORMATION

This study was part of the CINAPS randomized clinical trial, supported by the National Eye Institute of the National Institutes of Health NEI R01EY023261 to T.L.A.

## CONFLICT OF INTEREST STATEMENT

Authors disclose no conflict of interest.

## CLINICAL TRIAL REGISTRATION

This study is registered on ClinicalTrials.gov (Identifier: NCT03593031).

## PARTICIPANTS' CONSENT

A written informed consent was obtained from all participants.

## Data Availability

The data supporting this study's findings are available from the corresponding author upon reasonable request.
